# Simulated prism exposure in immersed virtual reality produces larger prismatic after-effects than standard prism exposure in healthy subjects

**DOI:** 10.1371/journal.pone.0217074

**Published:** 2019-05-24

**Authors:** Alexander A. Ramos, Emil C. Hørning, Inge L. Wilms

**Affiliations:** Department of Psychology, University of Copenhagen, Copenhagen, Denmark; University of Muenster, GERMANY

## Abstract

Previous studies have shown that the size of the leftward bias after exposure to rightward prism-deviation (the prismatic after-effect) depends on the degree of rightward prism-deviation as well as the type of visual feedback receives during exposure to prism-deviation.

In this study, we tested if it was possible to obtain a leftward bias in pointing precision using two different methods of creating diverted visual input by simulating a rightward prism diversion of visual input in immersive virtual reality. We compared the results to the leftward bias in pointing precision obtained after exposure to standard prism goggles deviating visual input 10 degrees to the right. Twenty healthy participants were subjected to one session of standard prism adaptation therapy under three different conditions of deviated visual input: 1) created by imitating a 10 degree leftward rotation of the head (VRR), 2) created by imitating a 2D leftward horizontal displacement of 10 degrees (VRS) and 3) a control condition using real right-deviating prisms (PCP). The study showed that the simulated prisms in the VRR and VRS conditions produced deviations in pointing precision of a similar size. However, exposure to the VRS and VRR conditions both produced larger prismatic after-effects than the exposure to real prism goggles. This research is important for the development and use of virtual reality systems in the rehabilitation of neglect after brain injury as it emphasizes that the adjustment to deviated visual input may be affected positively by the use of immersive virtual reality technology.

## Introduction

For more than a century, adaptation to the visual distortion created with prisms has been used to study experience-based plasticity in the visuomotor system. Stratton [1] found successful adaptation to a 180-degree deviation and since then, adaptations to other large and small prism-deviations have been used to test learning and adaptation in the perceptual and motor systems (e.g. [2–5]). In healthy subjects, the exposure to prism-deviation followed by removal of the prisms produces a prismatic after-effect, a deviation in pointing accuracy in the opposite direction to the prism-deviation [6]. The size of the after-effect correlates with the degree of distortion induced by prism goggles; the larger the deviation, the larger the size [7]. A surprising issue regarding the after-effect is that it does not equal the prismatic deviation. Usually, the after-effect produced is about 40% of the prism-deviation [3]; the reason for this is still an unresolved mystery.

Visuomotor adaptation to deviation of visual input based on trial-by trial visual error feedback is categorized as sensory-motor adaptation allowing the brain to modify existing motor patterns as needed [8]. The adaptation itself is theorized to be the sum of three separate error correction mechanisms: postural control, recalibration and strategic remapping [3]. Postural control is the active, conscious control of the limb movement once you have detected that targets are not being reached correctly. In other words, you consciously override the internal movement programme forcing your limb further towards the intended target. Recalibration is the recoding of spatially coded movement commands and strategic realignment is the process keeping the internal proprioceptive maps up to date on body position and size in relation to the outer world [9]. It has been demonstrated that the after-effect is an additive effect comprised of the effect of recalibration and strategic alignment [4, 10–12].

How and when feedback is provided during prism exposure affects adaptation. The size and composition of the after-effect depend to a certain degree upon whether or not the subjects are allowed to see the actual movement of the extremity during prism exposure [6, 13]. In pointing tasks, visual access to the full arm movement during exposure (concurrent access) produce less after-effect than access to only the terminal part of the movement towards a target (terminal exposure) [14–16]. The nature of the task being performed during exposure may also affect the size and type of adaptation and consequently the after-effect [17].

One of the unresolved questions is how the nature of terminal feedback during prism exposure influences the adaptation. Does seeing an actual finger during terminal exposure produce larger after-effects than seeing a representation of a finger like an image or a symbol?

In 2009, we developed a 2D PC version of the Prism Adaptation Therapy outlined by Frassinetti et al. [18] for experimental purposes. As we converted the paper-and-pencil therapy into advanced technology, we made changes to the way the subjects received feedback on pointing precision during the exposure phase. The original intent was to reduce the movement of physical equipment. Instead of seeing the position of their physical finger in relation to the fixed target (direct feedback) during exposure to prism-deviation, two conditions were tested. In the first, an “X” was shown on the terminal next to the fixed target (indirect feedback) and in the second the physical finger was shown [19]. Seeing ones finger directly produced larger after-effects than indirect feedback such as symbols [19].

In 2017, we developed an immersive virtual reality version of the PAT training in which we simulate prism-deviation. Knowing that visuomotor adaptation might be sensitive to changes in feedback, we initiated a study to verify if the immersive version of PAT in which the virtual positions of virtual fingers are being used as feedback would produce similar or different size after-effects in healthy subjects before testing the VR PAT injured subjects. Part of the study compared the after-effect produced by two different methods of simulating prism-deviation, a rotating and a skewing of visual input. This paper reports the results.

### Purpose

The purpose of this study was to test 1) if it is possible to produce a prismatic after-effect using simulated prism-deviation in immersive virtual reality, 2) whether the after-effect produced by simulated prisms in immersed virtual reality are smaller than the after-effect produced by prism goggles and 3) if there would be any difference in after-effect between the two types of simulated prism-deviation methods available in Unity.

Finally, the study also evaluated the potential benefits and drawbacks of using a virtual version of PAT.

### Theory

#### Virtual reality and the prismatic after-effect

Virtual reality (VR) is ‘an artificial environment which is experienced through sensory stimuli (such as sights and sounds) provided by a computer and in which one’s actions partially determine what happens in the environment’ [20]. This definition covers a range of methods to simulate and interact with a simulated world from a 2D presentation on a PC monitor to a total immersive visual, auditory and tactile environment [21, 22].

Recently, immersive virtual reality technology has become a resource in of cognitive rehabilitation as a challenging but safe and controlled training environment [23–25]. Immersive VR has proven to be a useful and constructive tool for assessment of cognitive deficits in executive functions [26] as well as skills such as driving [27] or shopping [28].

When immersed in a virtual world, the subject will usually be wearing a helmet or goggles covering the eyes and will have what seems like a direct interaction with the environment or objects through an avatar. Studies have shown that the brain easily adapts to virtual representations of arms and hands [29]. However, a very recent study has shown that different components of visuomotor adaptation are affected by conventional motor training and immersive virtual reality motor training despite similar surface behaviour [30]. As in the real world, the after-effect is large when using terminal exposure as opposed to concurrent exposure when adapting to a throwing task [31]. Deliberate misalignment of viewed hand position in immersed virtual reality produce no performance deviation but less after-effect [32]. Functional imaging studies of prism adaptation have demonstrated that manipulation of visual feedback of hand position in virtual reality may affect which brain areas being involved in the adaptation [33, 34].

Visuomotor adaptation is used in brain injury therapy to ameliorate the effects of unilateral neglect [2, 35], one of the more common deficits after brain injury to the right hemisphere [36]. Neglect is a syndrome commonly defined as a failure to explore, respond to or orient towards stimuli commonly presented in the left side of space [37]. Evidence supports the idea that visuomotor effects of unilateral neglect can be ameliorated by Prism Adaptation Therapy (PAT) [18, 35, 38, 39].

One common research paradigm used for testing prismatic adaptation consists of three steps. The first step is a pre-exposure/baseline step to measure the visuomotor accuracy of the subject (without prism goggles), usually established either by letting the subjects point out the subjective midline repeatedly (e.g. [35]) or by letting the subjects point to targets with the movement of their arm and hand disguised beneath a non-transparent barrier (blinded) [6, 18].

The second step is to expose the subjects to a visual distortion induced by prism goggles. Although subjects are fully aware of the fact that they are receiving distorted visual input, parts of the visuomotor system are not able to adjust to this knowledge. The result is that, initially, healthy subjects will tend to point off-target in the opposite direction of the prism-deviation at a distance depending on the prism displacement and direction [2]. At the end of each trial in the exposure step, subjects will receive feedback on pointing precision and gradually adapt to the prismatic change. As a result, the target precision will increase. Usually, subjects are able to adjust within a few trials, initially through conscious control (by forcing the hand to move further to the left than what seems natural) and after a while through more automated control [40]. As the motor control mechanism changes from conscious control to a level of more automated control, overcompensation can be observed for a brief period of time, causing a pointing deviation to the left of the target [10].

The third step is basically similar to the first step. Visual input is restored to normal by removing the prism goggles and the blinded pointing precision is re-measured.

## Material and methods

Twenty healthy subjects, 13 females of an average age of 25.9 (5.5) and 7 males of an average age of 28.3 (3.1) were tested under three different prism adaptation conditions: 1) a virtually simulated prism-deviation using virtual reality ROTATE (abbreviation VRR), 2) a virtually simulated prism-deviation using virtual reality SKEW (abbreviation VRS), and 3) prism-deviation using a standard set of prism goggles and PC (abbreviation PCP). All subjects were tested in all three conditions but assigned to one of four different sequences through randomisation to avoid learning effects. The four sequences were VRR-VRS-PCP, VRS-VRR- PCP, PCP-VRR-VRS and PCP-VRS-VRR. Subjects had a break of at least 10 minutes between each condition for adequate mental recovery.

In each of the three prism conditions, the participant completed 5 steps (Table 1).

**Table 1:** The 5 steps of each condition.

Step	Feedback	# of trials
1. Pre-test	No & Yes	9
2. Baseline (pre-exposure)	No	30
3. Exposure	Yes	90
4. Post-test (post-exposure)	No	60
5. Reset	Yes	30

In Table 1, the column ‘Feedback’ specifies whether the fingertip (virtual or real) would be visible right before hitting the target. ‘Trial’ indicates the number of pointing trials in total for the particular phase. The ‘Pre-test’ step includes the number of sample trials both with and without visible fingertip. The ‘Reset’ step was inserted to ensure that each subject left the test with a normalised visuomotor system. This was subsequently verified and confirmed through inspection of the captured raw data.

### Ethics

Subjects were provided with oral and written information before beginning the study about their rights, the purpose of the study and about the fact that they might experience temporary dizziness during the testing. They then signed written consent forms. All subjects went through a reset task to avoid any temporary deviation effects from the tests. The work was approved by the local ethical committee at the Department of Psychology.

### Equipment used

#### The PCP condition

The computer-based prism adaptation setup consisted of a PC, a touchscreen, a specially constructed wooden screen, prism goggles and a plastic tip to put on the subjects’ fingertip to increase pointing precision and protect the touch screen surface. The PC was a standard PC with Windows 7 installed. The attached touch screen was a 22” (477 mm wide) touch-sensitive TFT LCD monitor (DT220TSR5U) with a response time ≤ 5ms. The touch technology was a 5-wire, analogue resistive type with a touch resolution of 4096 x 4096 and a screen resolution of 1680 x 1050 pixels with a refresh rate of 75 Hz.

**Fig 1:**
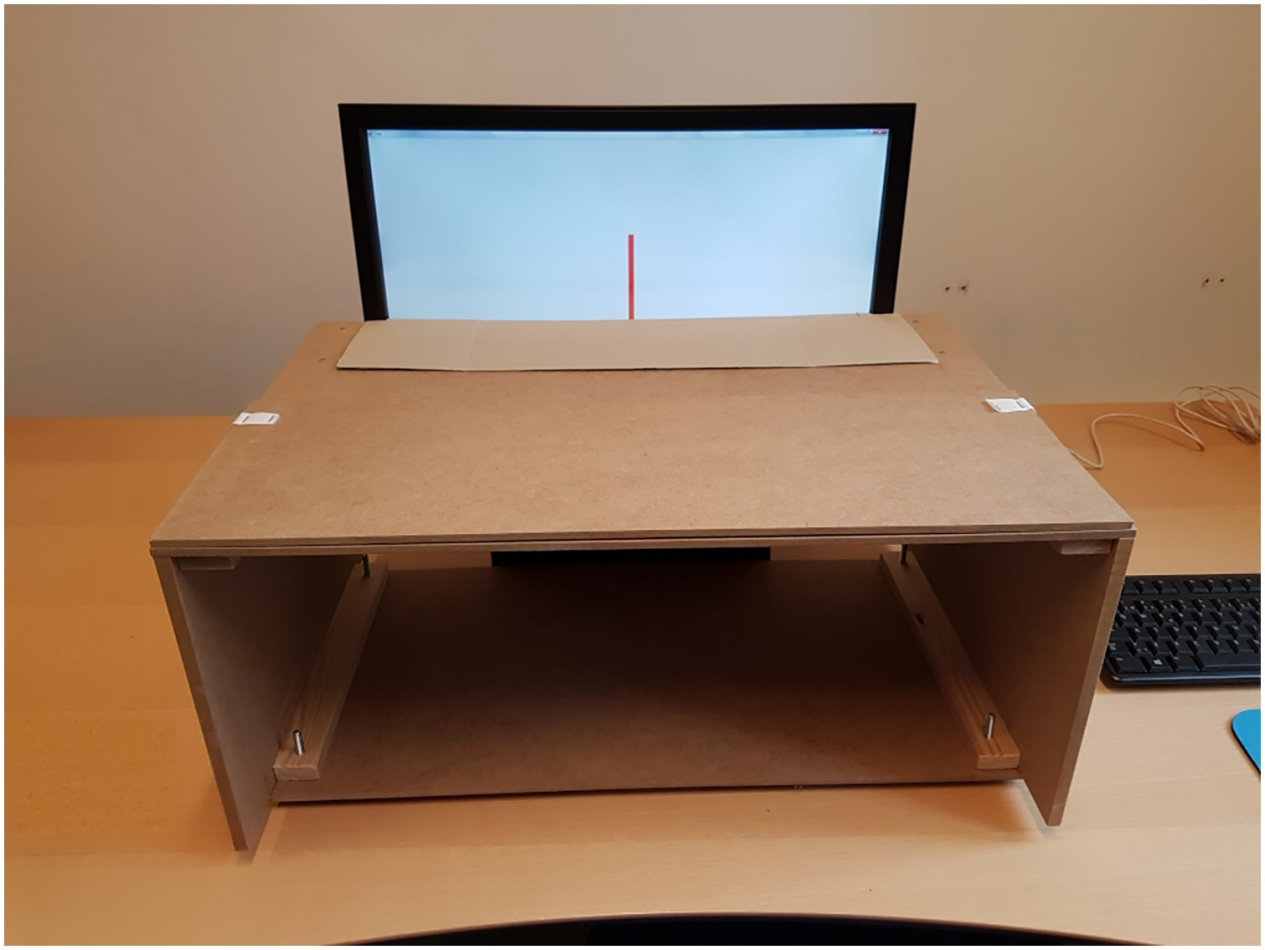
The PCP control session setup. The red line is the target.

The software programme used in the PCP condition was developed for a previous study by one of the authors, Inge Wilms [19]. Targets would appear at one of three different positions in the upper part of the touchscreen, one at the centre of the screen and one on each side of the centre at a distance of 528 pixels (= 14.5 degrees) (Fig 1). Along the same horizontal axis in a pseudo-random order controlled by an algorithm ensuring that each target was presented an equal number of times. The target would remain visible until the subject had responded. The programme recorded detailed information regarding accuracy of the subject’s pointing position throughout the session.

Only the top part of the touchscreen was visible to the subjects. A wooden screen in front of the touchscreen was used to reduce and control the subjects’ visibility of their arm movements and the touch area. The screen had a sliding top which was adjusted to the various arm lengths of the subjects. The touchscreen issued a beeping sound when touched, indicating to the subject that the pointing was recorded. The programme ignored any accidental repeated touches.

The prism goggles in this study were constructed using a standard pair of goggles with a large viewing area and lining them with Fresnel prisms.

#### The virtual reality conditions (VRR and VRS)

The HTC VIVE VR headset and controller were used in the study. A programme simulation of the PC environment was written in C# interfacing with Unity and SteamVR to interact with the graphics, the headset and the controller. This programme was developed by two of the authors (Ramos and Hørning). Data about the position of the targets and the touching precision were recorded in a log file on the PC. To record the precise position of the subject’s fingertip, the subject placed their fingertip on the ‘menu’ button on the controller.

**Fig 2:**
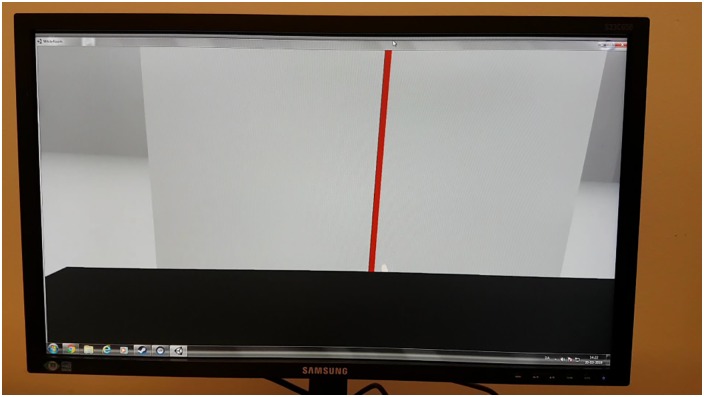
The VRx session setup. The red line is the target. The subjects would see the visual world in a head mounted device.

Only a graphic representation of a fingertip was visible to the subjects in the simulated environment, along with a white wall on which the targets were presented. A virtual black box concealed the virtual arm movements. In the exposure and reset tasks, the black box was positioned approximately 3 cm from the white wall, making the fingertip visible. In the VR conditions, the fingertip was programmed to be visible right below the top of the virtual black box regardless of where the subject pointed in the vertical plane. The HTC VIVE controller was programmed to rumble slightly when the white wall was touched in the VR simulation. This provided the subjects with an impression of actually hitting the target (Fig 2).

### Local-to-world matrix

Each object in a virtual world scene has a unique Local-to-World (LtW) matrix. The matrices are used to place and scale objects according to their position in the virtual world (VW). The camera in the VW is also represented by an LtW matrix but with the exception that the camera is assumed to be able to move as the centre object, causing all other objects to scale and reposition accordingly when the camera moves around.

The field of view from the perspective of the camera is termed the viewing frustum (VF) and specifies the boundaries of the objects in view. It has the shape of a frustum extending from the camera and consists of a near-plane and a far-plane. When a scene is being transposed from a 3D plane to a 2D plane for presentation, it is done using the VF. Objects very close and very far away are omitted for display. By changing aspects of the VF, it is possible to obtain different visual point-of-views. Changes in the VF were used in this study to simulate prism goggles in the following two different manners.

### The prism simulations

**Fig 3:**
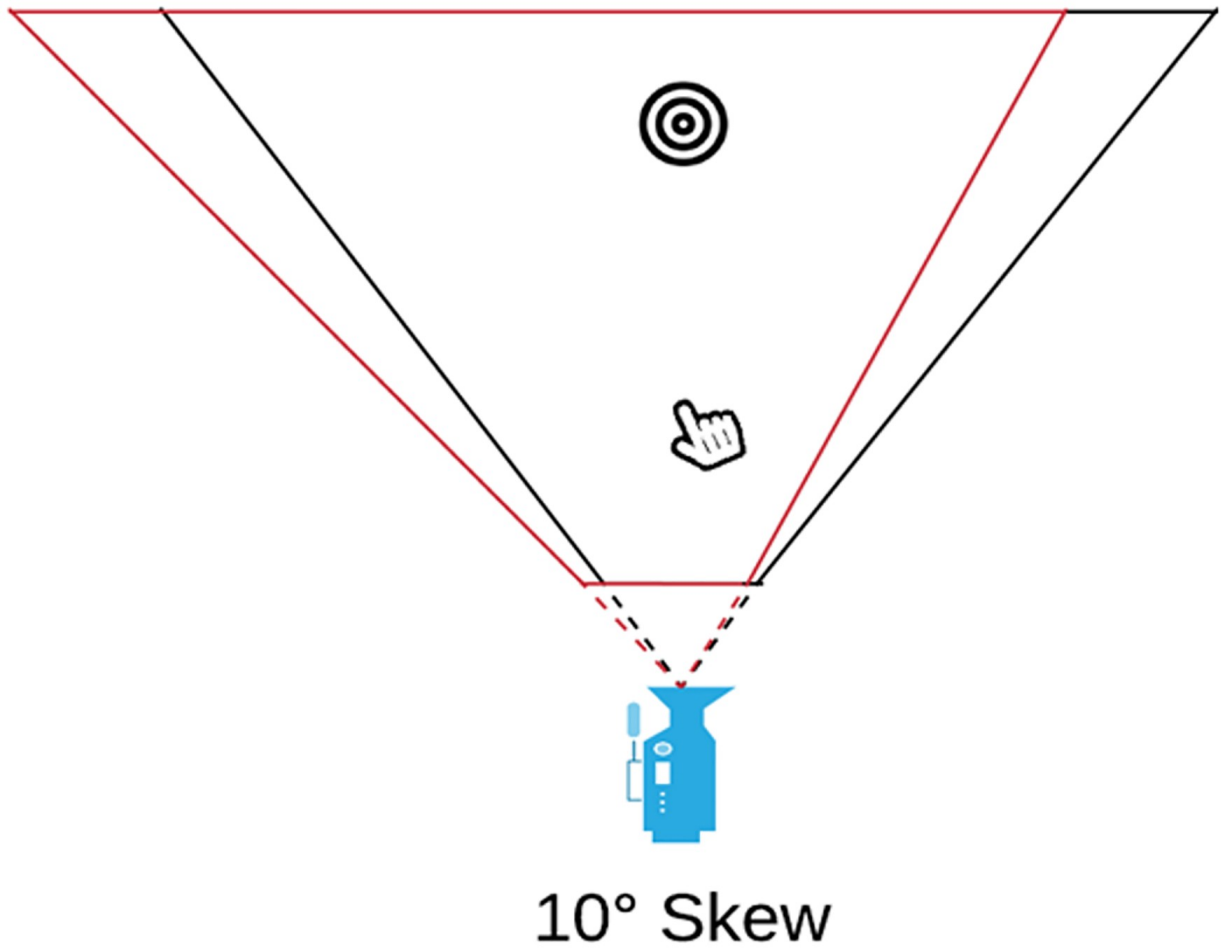
SKEW option. The near- and far-planes were skewed to the same side to simulate a 10-degree prism-deviation. Red line indicates the skewing.

With the SKEW prism simulation, the near and far planes were both skewed to the left by 10 degrees (Fig 3).

**Fig 4:**
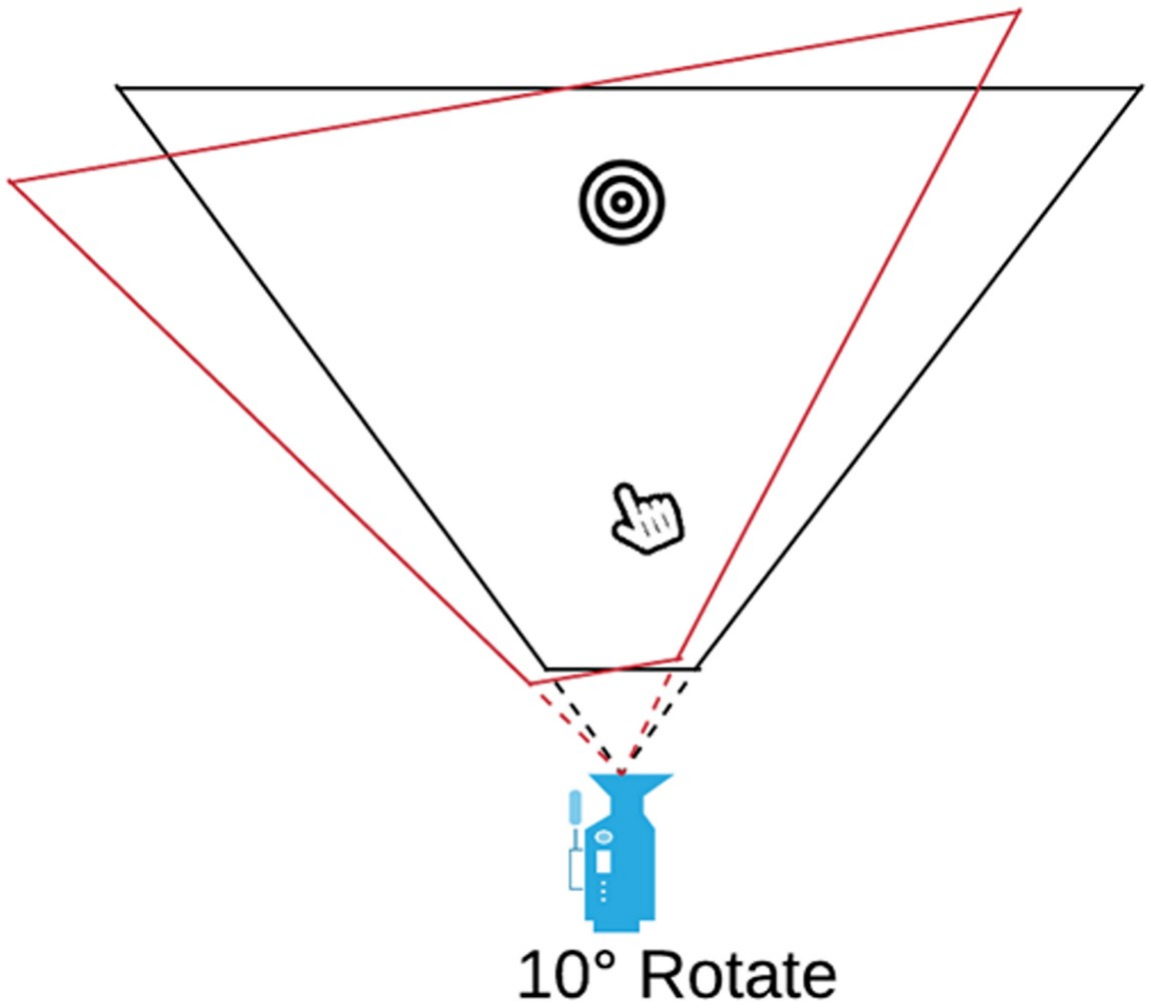
ROTATE option. The frustum was rotated to the left. The red line indicates the rotation.

With the ROTATE prism simulation, the frustum was rotated around the camera axis to the left (Fig 4).

### Statistics

A Linear Mixed Model (LMM) analysis was conducted using SPSS version 25.0 was used to analyse data using the following scripts:

MIXED Deviation by Condition Location Subject with PreAvg
/METHOD = REML
/PRINT = Solution
/FIXED Condition Location Condition*Location PreAvg
/RANDOM Subject Condition*Subject Subject*Location Condition*Location*Subject | Covtype(VC)
/REPEATED TrialNo | Subject(Subject*Condition) COVTYPE(AR1)
/EMMEANS Tables(Condition) COMPARE(Condition) ADJ(BONFERRONI)
/EMMEANS Tables(Location) COMPARE(Location) ADJ(BONFERRONI)
/EMMEANS Tables(Condition*Location) COMPARE(Condition) ADJ(BONFERRONI).

### Metrics

Data were captured in different metrics but were converted to deviations in degrees to allow comparison across conditions as well as across studies. In the conversion to degrees, the distance from touch position to target position in cm (a) along with arm length in cm (b) was used to calculate the angle of deviation in degrees (tan^-1^(a/b)*π).

### Outliers and adjustments

Data was inspected for extreme data. For the PCP condition, a total of 95 trials with 0 ms response time distributed randomly across all subjects were excluded as being artefacts of a bouncing finger.

Across subjects, seven trials which deviated above 3 SD from mean were excluded from the analysis in all three conditions. No subjects were excluded. In the PCP condition, the total percentages of excluded trial for the PRE and POST steps were 0.33%, and 1.25%. In the VRS condition, the percentages were 0.33% and 0% and in the VRR condition 0%, and 0%.

After completing the study, we discovered that the PCP condition had been conducted with an 8.70-degree prism-deviation and the virtual reality conditions with a 10-degree prism-deviation. It has been shown that there is a linear relationship between the degrees of prism-deviation and produced after-effect [7, 41] when doing similar tasks. We therefore decided to do a mathematical transformation of the results for the PCP condition from 8.70 degrees to 10 degrees to align the results for comparison and statistical analysis. In addition, we converted the transformed results to degrees and compared the results with data from a previous study using the same PC paradigm [See 19]. The mean and SD were similar in size between the old study and the transformed data. All data presented in the results section of the paper for the PCP condition is therefore post transformation data.

## Results

All scores are presented in degrees and are calculated as the difference from the centre of the displayed target to the actual pointing position. A negative value indicates a leftward pointing position and a positive value a rightward position relative to the centre of the target.

**Fig 5:**
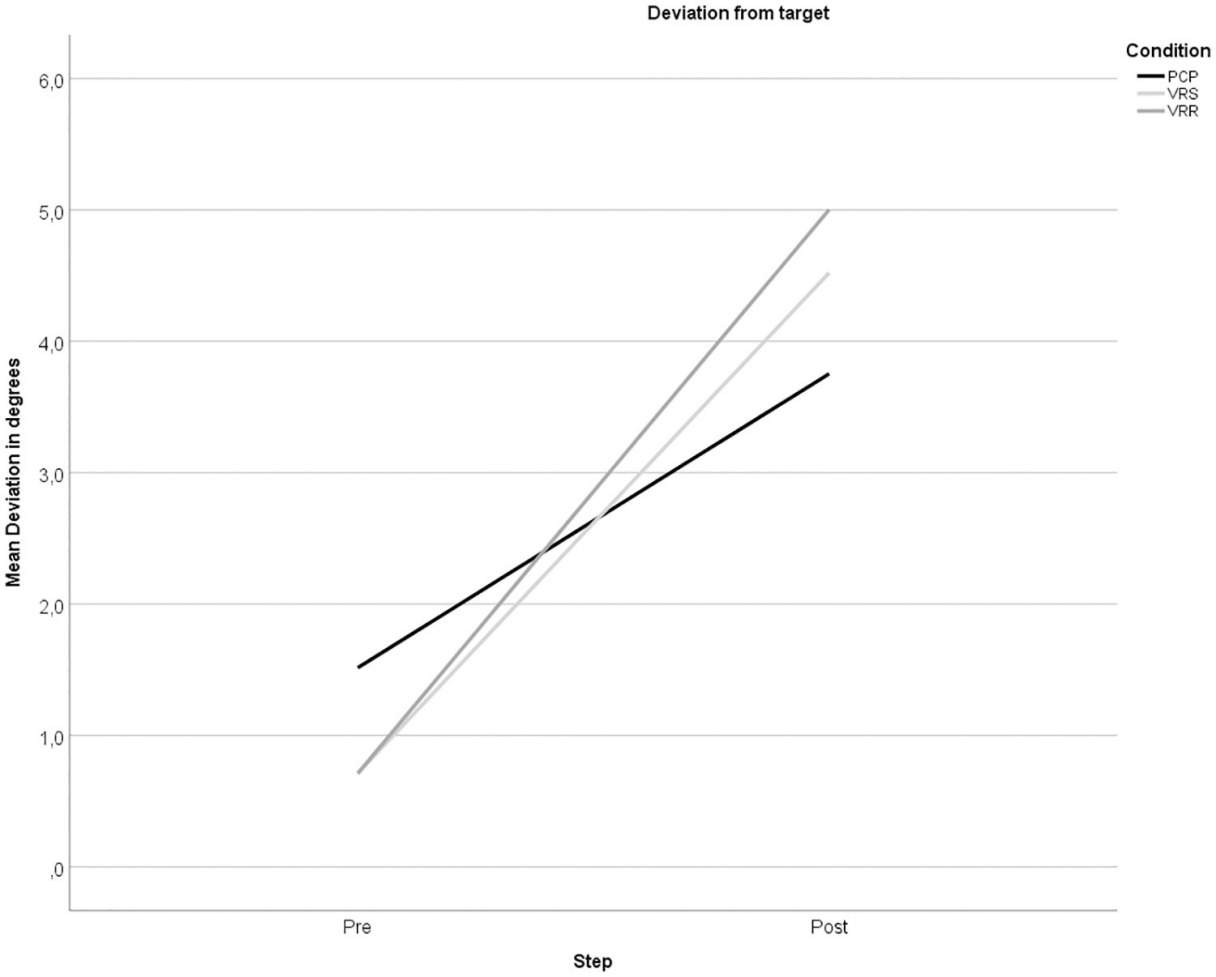
The slope for each condition from pre to post exposure in degrees.

Starting with a visual inspection (Fig 5), the mean deviations at pre and post showed a difference in after-effect between the three conditions. Further visual inspection of the data at subject level showed variation at both intercept and slope. For that reason, a LMM random slope and intercept model was used to test the difference between the three conditions. A Bonferroni correction was done to adjust for the repeated trial measures at condition and location level.

Two models were tested, one with pre exposure performance as a covariate and one without. The reason for this is that there are good theoretical reasons for inspecting both results. In the first case, it may be argued that subjects may have a prior lack of precision due to some invariant factors. If this is the case, it is necessary to adjust the resulting after-effect for any pre exposure deviations. However, it may also be argued that the 90 trial training during the exposure phase is adjusting the visuomotor system using live feedback. The exposure step itself is a reset of prior deviations and the resulting after-effect is therefore the pure deviation. The results from the two models were reasonable similar so only the results from the best fitting model, which included Pre exposure performance as covariate, is presented here. A post hoc Bonferroni adjustment was done to correct for repeated measures on trial and condition.

The analysis showed a fixed effect of condition *F* (2, 36.9) = 12.295, *p <*.005, location *F*(2, 37.4) = 15.1, *p* <.005, Pre *F*(1, 136.1) = 24.9, *p* <.005 but not the interaction condition*location *F*(4, 76.8) = 2.23, *p* = .068. Estimating marginal means with a pairwise comparison as part of the mixed model (Table 2) showed that the after-effect produced by the PCP condition deviated significantly from the two virtual reality conditions.

**Table 2:** Pairwise comparison of conditions with the after-effect adjusted for deviation prior to exposure.

Conditions		Mean Diff.	SE	df	p	CI Upper/Lower	
PCP	VRS	-1,120	0,328	37,429	0,005	-1,942	-0,298
	VRR	-1,588	0,328	37,450	0,000	-2,410	-0,766
VRS	PCP	1,120	0,328	37,429	0,005	0,298	1,942
	VRR	-0,468	0,323	35,985	0,466	-1,278	0,342
VRR	PCP	1,588	0,328	37,450	0,000	0,766	2,410
	VRS	0,468	0,323	35,985	0,466	-0,342	1,278

The parameter estimates from the model and the predictive effects is not presented as they have no relevance to the research questions.

## Discussion

The results confirm that it is possible to generate a prismatic after-effect in healthy subjects using immersive VR technology with simulated prism-deviation. Secondly, contrary to expectation, the after-effect produced with indirect feedback (you do not see your own finger but an image of a finger) in immersed virtual reality is larger than the after-effect produced by normal prism goggles (seeing your real finger in physical reality). Finally, the study demonstrated that there was no difference in the size of the prismatic after-effect between the two types of simulated prism-deviation available in Unity.

One major difference between the PCP and the VRx conditions was the box used to hide the arm movement. In the virtual reality conditions, the box followed the hand movement in the vertical plane, ensuring that the subject always saw the fingertip as feedback during the exposure step. In the PCP condition, the box was stationary, and although the assistants were monitoring correct behaviour, subjects would at times position their finger incorrectly and not receive feedback. This potential difference might explain a small part of the positive difference but probably not all.

### Limitations

There is a chance that the linear transformation of the PCP results may not reflect the results from exposure to a real 10-degree prism-deviation. However, the after-effects measured for this batch of subjects match the after-effects measured previously on the same paradigm with a 10-degree prism-deviation [See 19], strengthening the assumption that the virtual reality condition does in fact produce larger after-effects than those produced by ordinary prism goggles in standard conditions. Another theory might be that the subjects were less distracted during the exposure phase in the virtual reality conditions due to the use of the immersive headset. The heightened attentional focus might result in faster adaptation. However, a comparison between the learning curves for the three conditions showed no difference in the number of trials used to adjust to the deviations. On average, subjects required 7 to 9 trials to be able to point directly at the target during the exposure phase in all three conditions.

The exposure to indirect feedback may have a hidden influence on the choice and impact on the internal adaptation mechanisms. A greater after-effect does not in itself indicate the composition of the adaptation. Further studies are needed to confirm the durability and composition of the larger effect. It is still not fully understood why the measured after-effect is approximately 40–50% of the prism-deviation despite flawless pointing precision during exposure [3].

### Considerations on the use of immersive VR

The test in the study was intentionally done in a similar fashion to a traditional session of prism adaptation therapy [35]. Although the VR equipment required initial calibration, the potential use in PAT is interesting as it may allow for further optimisation of training for several reasons. PAT basically depends on the detection of a visuomotor discrepancy. In a physical environment, it can be difficult to provide correct feedback to the subjects because the movements of the arm and hand must be hidden up until hitting the target. In our experience, subjects often fail to keep the hand in a position which allows them to receive terminal feedback every time they point to a target. The terminal feedback of the finger is important for the activation of the correct adaptive mechanisms [6, 15, 16].

In the VR conditions, the flexible frame concealing the arm movement ensured that the subjects received correct terminal feedback during all exposure trials. Moreover, the VR system facilitates tracking of the speed and position of the arm at any time which enables full control of the timing of the appearance of the target in relation to the arms being fully retracted. Our current implementation did not provide a speed warning for too slow or fast movement in this implementation, but it would be easy to verify the speed of the movement online and provide ample and automatic warnings alerting the subjects to move faster or slower. All three points are challenges which normally would require constant human supervision during prism adaptation training.

The subjects did not report any dizziness or nausea during the prism-deviation sessions. They did, however, comment on the weight of the head mount. Another challenge using the current virtual reality equipment is the requirement to use the quite heavy controller when completing the pointing activity. Several subjects had to take small breaks because of fatigue in their arms. If this type of training is to develop further into a clinical practice, it is necessary to develop a small unit or glove which tracks the finger position of the subjects. A final challenge is the current requirement for a very high-speed graphics unit on the PC despite the fact that the graphics used in the study were simple. It currently prevents the use of a portable laptop PC to be used in a clinical setting or for home training.

It still remains to be seen what the adaptation effect will be on real neglect patients and for more than one session. This will be the next area of research.

## Conclusion

We wanted to investigate if it was possible to produce a prismatic after-effect using simulated prisms in virtual reality. We also wanted to know if the after-effect from simulated prisms would be smaller or larger than the after-effect produced by normal prism goggles. Finally, we wanted to compare the effects of two different types of simulated virtual prisms. The results show that the two types of simulated prism-deviation produce larger after-effects than that produced by real prism goggles in healthy subjects. The study also showed that the two different methods of simulating prism-deviation in virtual reality produce after-effects of equal magnitude in healthy subjects.
