# Correction: Simulated prism exposure in immersed virtual reality produces larger prismatic after-effects than standard prism exposure in healthy subjects

**DOI:** 10.1371/journal.pone.0314790

**Published:** 2024-11-27

**Authors:** Alexander A. Ramos, Emil C. Hørning, Inge L. Wilms

The Data Availability statement for this paper is incorrect. The correct statement is: All raw data files are available from ResearchGate at the following URL: https://www.researchgate.net/publication/353175627_Trialdata_for_VRRVRSPCPstudy_all_subjectssav?channel=doi&linkId=60ebf4fcb8c0d5588cee79bb&showFulltext=true; DOI: 10.13140/RG.2.2.35523.32805.
